# The Kynurenine Pathway, Aryl Hydrocarbon Receptor, and Alzheimer’s Disease

**DOI:** 10.3390/brainsci14090950

**Published:** 2024-09-23

**Authors:** Enoc Mariano Cortés Malagón, Adolfo López Ornelas, Irlanda Olvera Gómez, José Bonilla Delgado

**Affiliations:** 1 Research Division, Hospital Juárez de México, Mexico City 07760, Mexico; emcortes@cinvestav.mx (E.M.C.M.); adolfolopezmd@gmail.com (A.L.O.); irlandaolverag@gmail.com (I.O.G.); 2 Genetics Laboratory, Hospital Nacional Homeopático, Mexico City 06800, Mexico; 3 Facultad Ciencias de la Salud, Universidad Anáhuac Norte, Estado de México 52786, Mexico; 4 Research Unit, Hospital Regional de Alta Especialidad de Ixtapaluca, IMSS-BINESTAR, Ixtapaluca 56530, Mexico

**Keywords:** Alzheimer’s disease, neuroinflammation, kynurenine pathway, AhR

## Abstract

Alzheimer’s disease (AD) is the leading cause of dementia, mainly affecting elderly individuals. AD is characterized by β-amyloid plaques, abnormal tau tangles, neuronal loss, and metabolic disruptions. Recent studies have revealed the involvement of the kynurenine (KP) pathway and the aryl hydrocarbon receptor (AhR) in AD development. The KP pathway metabolizes tryptophan to produce neuroactive substances like kynurenine, kynurenic acid, and quinolinic acid. In AD, high levels of kynurenine and the neurotoxic quinolinic acid are associated with increased neuroinflammation and excitotoxicity; conversely, reduced levels of kynurenic acid, which acts as a glutamate receptor antagonist, compromise neuroprotection. Research has indicated elevated KP metabolites and enzymes in the hippocampus of AD patients and other tissues such as blood, cerebrospinal fluid, and urine. However, the finding that KP metabolites are AD biomarkers in blood, cerebrospinal fluid, and urine has been controversial. This controversy, stemming from the lack of consideration of the specific stage of AD, details of the patient’s treatment, cognitive deficits, and psychiatric comorbidities, underscores the need for more comprehensive research. AhR, a ligand-activated transcription factor, regulates immune response, oxidative stress, and xenobiotic metabolism. Various ligands, including tryptophan metabolites, can activate it. Some studies suggest that AhR activation contributes to AD, while others propose that it provides neuroprotection. This discrepancy may be explained by the specific ligands that activate AhR, highlighting the complex relationship between the KP pathway, AhR activation, and AD, where the same pathway can produce both neuroprotective and harmful effects.

## 1. Introduction

Alzheimer’s disease (AD) is the most common cause of dementia, accounting for 60 to 70% of cases worldwide. It is estimated that approximately 10% of individuals over the age of 65 are affected by AD, with an increase of 32% among those aged 85 and older, with the annual incidence of AD estimated at 6.48% [1]. The global population of individuals with AD and related dementias (ADRDs) has reached approximately 51.62 million. These diseases are more frequent in females (32.62 million) than in males (18.7 million) [2]. The prevalent case rate in 2021 varied by country, with the highest prevalence per 100,000 cases in Japan (3079), Italy (2269), Slovenia (1963), Monaco (1962), and Greece (1874). The highest prevalence by continent per 100,000 cases was in Europe (1443), America (938), and Asia (598) [3]. By 2050, there will be 152 million cases of these diseases worldwide [4]. The high prevalence of ADRDs is believed to be the result of a combination of factors, including longer life expectancy, genetic predispositions, and certain lifestyle factors common in these regions [1].

The three most prevalent risk factors associated with ADRDs worldwide are as follows: Europe: physical inactivity (20%), low education (14%), and smoking (14%); Latin America: low education (29%), midlife hypertension (25%), and midlife obesity (21%); Australia: physical inactivity (20%), midlife obesity (17%), and low education (15%); China: low education (31%), physical inactivity (23%), and midlife hypertension (19%); Mozambique: physical inactivity (31%), low education (21%), and smoking (11%) [5].

Individuals with AD typically experience a gradual decline in cognitive abilities, including memory loss, language difficulties, executive dysfunction, and impaired visuospatial skills. In the early stages, mild forgetfulness and occasional disorientation are common, which worsen over time, leading to increased confusion, mood changes, and a significant decline in the ability to perform daily tasks. As the disease progresses, individuals often require full-time care and experience noticeable reductions in their ability to communicate, recognize familiar people, and carry out routine activities independently [6].

In addition to these clinical symptoms, AD involves complex biochemical abnormalities, such as amyloid-β plaques, neurofibrillary tangles composed of hyperphosphorylated tau protein, oxidative stress, and herpes simplex virus infections [6,7].

AD comprises two genetically distinct forms: early-onset or familial AD and late-onset or sporadic AD. The first one, accounting for <1% of AD cases, is caused by rare and fully penetrant mutations in three genes: amyloid-β precursor protein (APP), presenilin 1 (PS1), and presenilin 2 (PS2). The second one is the most common form of the disease; nevertheless, both forms of AD exhibit similar clinical pathology [8].

The Aβ plaques consist of extracellular aggregates; these peptides typically range from 39 to 42 amino acids in length and are generated through sequential cleavages of APP by β and γ-secretases. Aβ40 is the predominant form of Aβ peptide, secreted and retrieved from cerebrospinal fluid, while the Aβ42 peptide constitutes less than 10% [9]. However, the most amyloidogenic Aβ peptide in AD is Aβ42, and it has been observed to be the initial and predominant peptide found in the plaques [10]. Oligomeric Aβ42 has been shown to interfere with synaptic function by disrupting synaptic signaling and plasticity, leading to synaptic loss well before neurons undergo apoptosis or necrosis. In vitro and in vivo models of AD demonstrate that Aβ42 oligomers impair long-term potentiation (LTP), a cellular mechanism underlying memory formation, thereby contributing to early cognitive impairments [8].

On the other hand, tau is a member of the microtubule-associated protein family and is primarily located in axons, with effects on neuronal development, vesicular and axonal transport, and neuronal polarity [11]. Physiologically, tau is phosphorylated or dephosphorylated in a balanced state, enabling stable tubulin polymerization [12]. Hyperphosphorylation induces oligomerization and filament formation, forming neurofibrillary tangles (NFTs), leading to cytoskeletal disturbances with repercussions on axonal transport synapses and resulting in neuronal death [13].

Excessive stimulation of neurons by certain neurotransmitters, particularly glutamate, leads to excitotoxicity, a key factor in AD. This process involves overactivation of glutamate receptors—N-methyl-D-aspartate (NMDA) and α-amino-3-hydroxy-5-methyl-4-isoxazolepropionic acid (AMPA) receptors. This overactivation leads to an abnormal influx of calcium ions, triggering harmful events that contribute to cellular damage and death [14]. After releasing glutamate in the synapse, astrocytes control overstimulation of the receptors by taking them up via the excitatory amino acid transporters (EAATs). Studies have observed a decrease in glutamate transport in the cortex and hippocampus of AD patients, which is attributed to a downregulation in the expression of EAAT1 and EAAT2 [15,16]. Notably, neurons near Aβ plaques exhibit elevated excitability [17]. In cultured cell models, Aβ42 has been shown to promote the release of the NMDAR co-agonist D-serine from microglia and increase glutamate release from astrocytes [18]. The relationship between tau and neuronal hyperexcitability remains controversial. For instance, mutations in the MAPT gene that encodes tau have been linked to elevated excitability in neuronal networks [19]. In transgenic mice, overexpression of human tau with the A152T mutation resulted in elevated extracellular glutamate levels and increased neuronal loss [20]. Furthermore, tau may also lead to hypoexcitability; transgenic mice expressing human tau with the P301L mutation exhibited reduced neuronal activity. Tau may also reduce neuronal excitability by affecting the axon initial segment (AIS) where action potentials start [21]. Hyperphosphorylated tau destabilizes microtubules, causing the AIS to move away from the soma and reducing neuronal firing frequency. Different tau phosphorylation sites have various effects: hyperphosphorylation of T231 (AT180E) and S262/S356 (12E8) results in AIS relocation, while S396/404 (PHF1) does not impact AIS positioning [22].

Based on the temporal occurrence of Aβ and tau pathologies and the evidence that Aβ overproduction leads to AD, the amyloid cascade hypothesis was proposed, posing that its accumulation is the primary event triggering a cascade of effects resulting in neuronal damage [7]. However, increasing evidence suggests that the amyloid cascade alone cannot account for a substantial portion of AD pathogenesis. Research on the spatiotemporal progression of AD has shifted focus from Aβ plaques to NFTs as a central element in disease pathology. Studies by Braak and colleagues have demonstrated that the distribution of tau pathology, which leads to the formation of NFTs, correlates more directly with cognitive decline than Aβ plaques. Their work revealed that the progression of tau pathology follows a clear and predictable pattern, better matching the stages of clinical deterioration in AD than the accumulation of Aβ plaques [23,24]. Although the association between A tau and AD has been extensively studied, the role of other cofactors should not be ignored.

Mitochondrial dysfunction has been identified as a leading cause of oxidative stress in AD. Mitochondria are crucial for cellular respiration, but they can be damaged by excess oxidative stress; this leads to the production of reactive oxygen species (ROS), further damaging the cells. ROS can cause an increase in the production and accumulation of Aβ due to APP cleavage in AD. Furthermore, damage to the mitochondria hinders energy metabolism, contributing to synaptic dysfunction and cognitive decline [25]. In addition to oxidative stress, emerging evidence highlights the role of dysregulated glucose metabolism in AD. Brain glucose hypometabolism, often detected decades before clinical symptoms appear, has been closely linked to both amyloid and tau pathologies. Glucose uptake is reduced in key brain areas such as the hippocampus and frontal cortex, further accelerating neuronal dysfunction. This reduction in glucose metabolism leads to impaired ATP production, which is essential for maintaining synaptic function and neural plasticity [26,27].

Furthermore, dysregulated lipid metabolism, particularly cholesterol homeostasis, has been associated with AD pathogenesis. Cholesterol is critical for maintaining synaptic integrity, and its imbalance has been linked to the accumulation of amyloid plaques. Studies have shown that higher cholesterol levels can promote the formation of Aβ plaques. At the same time, specific cholesterol-lowering interventions have been found to reduce Aβ levels and improve cognitive function in AD models [28,29]. Apolipoprotein E (ApoE) variant E4 has also been identified as a significant genetic risk factor for AD [30]. Furthermore, reduced levels of docosahexaenoic acid, an omega-3 fatty acid, have been observed in the hippocampus region of the brains of AD patients [31].

Vascular factors have also been identified as significant contributors to AD pathogenesis. Studies have revealed that reduced blood flow and cerebrovascular damage contribute to the breakdown of the blood–brain barrier (BBB), enabling harmful substances to enter the brain and promoting neuroinflammation. Vascular risk factors such as hypertension and diabetes (T2DM) have been associated with an increased risk of developing AD [32,33].

Increasing evidence suggests that pathogens may have a role in AD development. It is suggested that specific pathogens can lead to the accumulation of Aβ1–42 monomers and the hyperphosphorylation of tau proteins by inhibiting the enzymes GSK3β and PKA or triggering a pro-inflammatory response. Studies using 3D human brain-like cultures have observed that cells infected with HSV-1 release pro-inflammatory mediators associated with AD, such as IL-1β, IL-6, and IFN-γ [34].

Neuroinflammation plays a critical role in the development of AD. Infection, trauma, ischemia, and toxins can produce pro-inflammatory cytokines. Releasing these molecules can cause synaptic dysfunction, neuronal death, and inhibition of neurogenesis [35,36].

The neuroinflammation and kynurenine pathway (KP) are closely connected and play a fundamental role in various neurological conditions [37]. The KP is a significant metabolic pathway involving the conversion of tryptophan (Trp) into a series of compounds, some of which have neuroactive effects [38]. Inflammatory factors can influence the KP enzymes’ activity, enhancing this pathway’s activity [39].

The KP has been observed to be dysregulated in AD patients. For instance, an increase in IDO1 activity has been reported in brain regions implicated in the disease, such as the hippocampus. Additionally, levels of kynurenine (Kyn) and other pathway metabolites may be imbalanced in AD [40].

Another factor related to the KP is the aryl hydrocarbon receptor (AhR). The AhR is present in various immune system cells, including microglia, a component of immune cells in the brain. AhR activation can modulate the immune response and inflammation [41]. This activation in CNS cells can influence cell survival, inflammation, and neuronal function. These effects may be relevant to the neurodegeneration observed in AD. Some compounds, such as kynurenic acid (KA) and quinolinic acid (QA), derivatives of the KP (discussed later), can be agonists [21] and antagonists [42] of AhR, respectively. Furthermore, it has been suggested that AhR may influence the production and balance of the KP and its metabolites. Given the association of the KP with AD and other neurodegenerative diseases, the interaction between AhR and these compounds could affect disease pathogenesis.

This review explains how the KP and AhR activation are interconnected in AD.

### 1.1. The KP in Mammals

**Figure 1:**
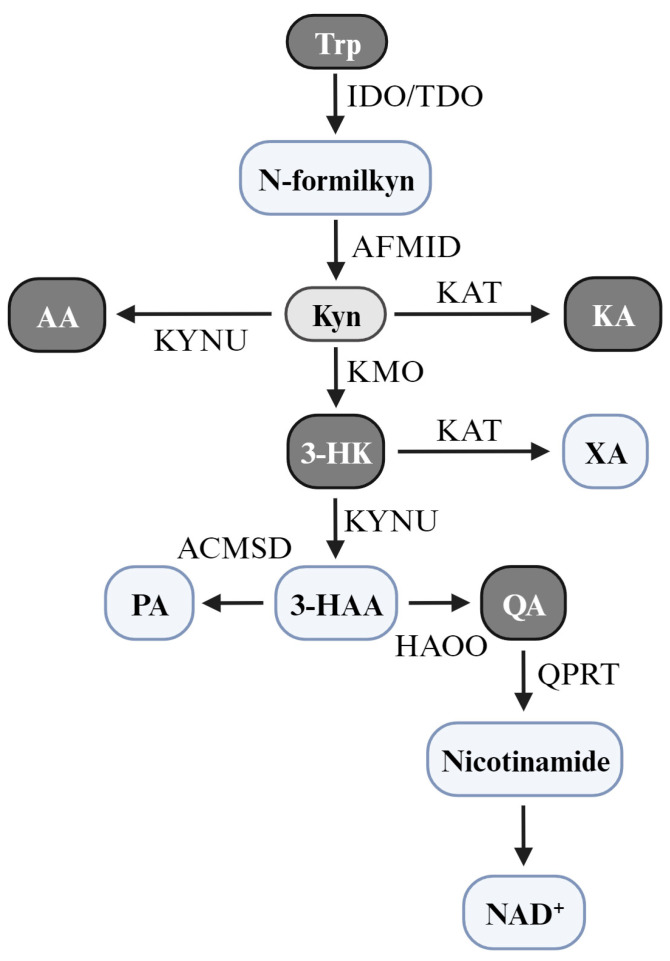
The kynurenine pathway. The pathway commences with the conversion of tryptophan to N-formyl kynurenine, which is subsequently converted to kynurenine. This metabolite is pivotal as it initiates a series of three reactions leading to the formation of various metabolites, including anthranilic acid, 3-hydroxy anthranilic acid, and kynurenic acid. These metabolites serve diverse biological functions, including regulating the immune system and synthesizing crucial coenzymes such as NAD+. Trp, tryptophan; IDO, indoleamine 2,3-dioxygenase; TDO, tryptophan 2,3-dioxygenase; N-formylkyn, N-formyl kynurenine; AFMID, kynurenine formamidase; Kyn, kynurenine; KYNU, kynureninase; AA, anthranilic acid; KAT, kynurenine aminotransferases; KA, kynurenic acid; KMO, kynurenine monooxygenase; 3-HK, 3-hydroxykynurenine; XA, xanthurenic acid; 3-HAA, 3-hydroxyanthranilic acid; ACMSD, α-amino-β-carboxymuconate-ε-semialdehyde decarboxylase; PA, picolinic acid; HAOO, 3-hydroxy anthranilate 3,4-dioxygenase; QA, quinolinic acid; QPRT, quinolinate phosphoribosyltransferase; NAD+, nicotinamide adenine dinucleotide.

The KP begins with the enzymatic conversion of 95% dietary Trp into N-formyl kynurenine. Approximately 90% of Trp is metabolized in the liver by the enzyme tryptophan 2,3-dioxygenase 2 (TDO2), which regulates systemic tryptophan levels. About 5–10% is metabolized by the enzymes IDO1 and IDO2 in immune cells, microglia, astrocytes, and neurons [43]. Subsequently, N-formyl kynurenine undergoes enzymatic conversion (formamidase/kynurenine formylase) to form kynurenine (Kyn) [44]. The Kyn metabolite can follow three metabolic pathways. The first metabolic pathway is catalyzed by kynurenine monooxygenase (KMO), which adds an -OH group to Kyn, forming 3-hydroxy kynurenine (3-HK). Subsequently, 3-HK is oxidized by kynureninase (KYNU) to form 3-hydroxy anthranilic acid (3-HAA). The 3-HAA is a 3-hydroxy anthranilate 3,4-dioxygenase (HAOO) substrate, which produces quinolinic acid (QA). QUIN produces the final metabolite nicotinamide adenine dinucleotide (NAD+), catalyzed by quinolinate phosphoribosyl transferase (QPRT) [45]. In addition, from 3-HAA, the ACMS decarboxylase produces picolinic acid (PA). In the second pathway, Kyn can be transaminated by kynurenine aminotransferases (KAT I–KAT III) to form kynurenic acid (KA) and xanthurenic acid (XA). A third route for Kyn involves its breakdown by kynureninase (KYNU), leading to the formation of anthranilic acid (AA), which is further metabolized by KMO, forming 3-hydroxy anthranilic acid (3-HAA) [46] (Figure 1).

The KP enzymes and metabolites are present in different mammalian tissues and cells, and their expression is tightly regulated, mainly by immune system mediators [47]. As mentioned, IDO1 is found in various immune and non-immune cells [43]. In the CNS context, IFN-γ is the predominant cytokine implicated in the induction of IDO1 in macrophage/microglial/dendritic cells [48]. Furthermore, TNF-α and IL-6 can also activate the expression of IDO1 in a manner that is independent of IFN-γ [49]. LPS can induce IDO1 expression in the cortex and hippocampus via IL-1β, TNF-α, and IL-6 [50,51]. Concerning the effects of anti-inflammatory cytokines IL10 and IL4 on IDO1 induction in CNS cell lines, it has been observed that IL-10 inhibits the induction of IDO1 mRNA mediated by IFN-γ in GT1-7 cells (mouse hypothalamic neuronal cell line) [52]. Conversely, in the EOC13.31 mouse microglia cell line, it was found that IL-4 enhanced, rather than suppressed, IFN-γ-induced IDO1 mRNA expression [53].

Inflammatory mediators also regulate the production of other enzymes within the KP [47,54], although there is limited research on these effects compared to IDO1. Specifically, LPS and IL-1β may upregulate KMO and KYNU enzymes in the rat brain and human hippocampal progenitor cells [50,51]. Contrarily, LPS does not affect KAT II expression in the rat brain, and IL-1β reduces KAT I and KAT III mRNAs in human hippocampal progenitor cells [48,51]. In addition to cytokines, KP regulation is modulated by other cellular stressors and environmental factors that impact its activity. For instance, bacterial products and chemical agonists of Toll-like receptors (TLRs), including TLR-2, TLR-3, TLR-4, TLR-7/8, and TLR-9, have been shown to induce KP activation in human monocytes, with TLR-3 stimulation specifically leading to increased levels of KA and quinolinic acid (QA) [55]. Furthermore, reactive oxygen species (ROS) are another trigger for KP activation, as oxidative stress has been reported to upregulate IDO1 expression [56]. Also, some metabolites of the KP can function as inhibitors of its enzymes. For instance, AA is an inhibitor of HAOO enzymatic activity, leading to decreased QA from 3-HAA [57].

The metabolites of the KP have diverse and often opposing roles, and they are crucial to a wide range of biological processes. In health, KP metabolites contribute to neuroprotection, immune tolerance, and energy production, while in disease states, they can drive neuroinflammation, excitotoxicity, and oxidative damage. For example, Kyn is an aryl hydrocarbon receptor (AhR) ligand that plays a significant role in immune regulation; it can suppress T cell proliferation and promote immune tolerance by enhancing the production of regulatory T cells (Tregs) [58]. It also inhibits the activation of effector T cells and natural killer (NK) cells, contributing to immune suppression, especially in the tumor microenvironment [59,60]. KA also has immunomodulatory properties. It has been shown to exhibit anti-inflammatory effects in inflammatory conditions and is relevant for maintaining the immunosuppressive microenvironment in many types of cancers. KA acts as a ligand for the GPR35 and the AhR receptors, inhibits the MAPKK signaling pathway, and plays a significant role as an antioxidant and scavenger of ROS (as mentioned later) [61].

Furthermore, as mentioned later, metabolites such as KA, 3-HK, and QA are crucial in understanding the balance between neuroprotection and neurotoxicity and their roles in CNS disorders.

### 1.2. Metabolism and Neuroactive Properties of Kynurenine in the Brain

**Figure 2:**
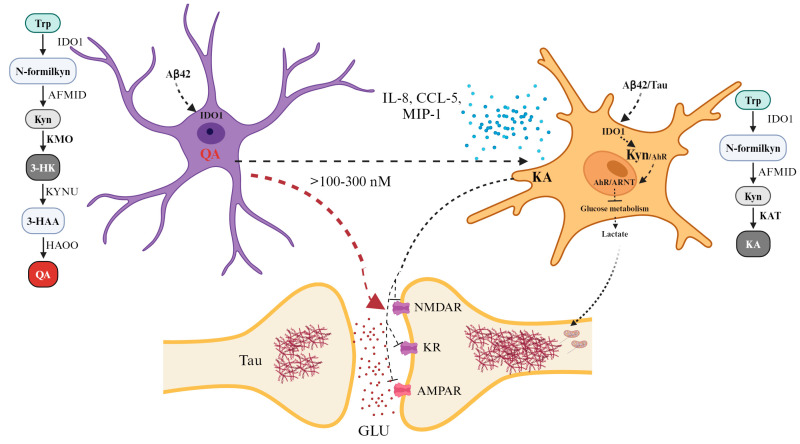
Neurotoxic effects of kynurenine pathway metabolites. KP metabolism begins with substrates Trp, Kyn, and 3-HK derived from the bloodstream or formed locally. Astrocytes are characterized by the expression of the KAT enzyme, resulting in the production of KA. KA acts as an antagonist of NMDAR, KAR, and AMPAR [63]. On the other hand, microglia express the KMO enzyme, leading to the production of QA. The latter is a weak agonist of NMDARs. In AD, Aβ42 induces the expression of IDO1, increasing QA levels, leading to NMDAR overexcitation and causing neurotoxicity due to the exacerbated release of GLU [64]. QA also induces the expression of proinflammatory cytokines (IL-8, CCL-5, and MIP-1), tau phosphorylation, and apoptosis. Aβ42 and tau increase Kyn production via overactivation of IDO1 in astrocytes. Kyn binds to AhR, translocates to the nucleus, and diminishes glucose metabolism. KP, kynurenine pathway; Trp, tryptophan; Kyn, kynurenine; 3-HK, 3-hydroxykynurenine; NMDAR, N-methyl-D-aspartate receptor; KAR, kainate receptor; AMPAR, α-amino-3-hydroxy-5-methyl-4-isoxazole receptor; KAT, kynurenine aminotransferases; KA, kynurenic acid; KMO, kynurenine monooxygenase; QA, quinolinic acid; GLU, glutamate; AhR, aryl hydrocarbon receptor; ARNT, AhR nuclear translocator.

The metabolism of this pathway in the brain utilizes Trp, Kyn, and 3-HK as substrates, originating from both the bloodstream and locally formed metabolites. Approximately 60% of KP metabolites in the CNS are derived from peripheral Kyn transported through the long and neutral amino acid transporter (LAT1, SLC7A5) across the blood–brain barrier (BBB) [62]. Functionally, two pathway branches are physically segregated in the brain (Figure 2). Astrocytes, which harbor the enzyme kynurenine aminotransferase (KAT), do not contain kynurenine monooxygenase (KMO) and, therefore, cannot produce 3-hydroxykynurenine (3-HK) from kynurenine (Kyn). Instead, they produce kynurenic acid (KA), which acts as an antagonist of glutamate receptors, inhibiting all three ionotropic excitatory amino acid receptors (NMDARs, kainate receptors, and AMPA receptors), and serves as a negative allosteric modulator of the α7 nicotinic acetylcholine receptor (α7nAChR) [63]. KA has also been shown to act as an agonist of the G protein-coupled receptor GPR35, modulating cAMP production and inhibiting N-type calcium channels in sympathetic neurons and astrocytes, thus deactivating inflammatory pathways. Additionally, KA possesses antioxidant properties related to its ability to scavenge hydroxyl radicals, superoxide anions, and other free radicals [61]. Conversely, in activated resident microglia and infiltrating macrophages, kynurenine monooxygenase (KMO) metabolizes kynurenine, leading to increased production of 3-HK, 3-hydroxy anthranilic acid (3-HAA), and quinolinic acid (QA).

The 3-HK molecule plays various physiological roles. At neutral pH, it self-oxidizes to form o-semiaminoquinone, which reacts with oxygen, generating o-aminoquinone and many O_2_^−^ and H_2_O_2_ radicals [64]. Moreover, it stimulates glutathione S-transferase, superoxide dismutase, and the transcription factor nuclear factor erythroid-derived 2-like 2 (Nrf2), which is crucial for antioxidant regulation. The 3-HK molecule is found at nanomolar concentrations in CNS in normal conditions, but at high concentrations, 3-HK is associated with oxidative activity [65]. Similar to 3-HK, 3-HAA has both pro-oxidant and antioxidant properties. 3-HAA functions as an anti-inflammatory and neuroprotective molecule by upregulating heme oxygenase-1 and inhibiting the cytokine and chemokine production induced by IL-1, IFN-γ, and TLR ligands, thereby contributing to neuroprotection [66]. It is also reported that 3-HAA inhibits the aggregation of Aβ plaques in a dose-dependent manner in vitro [67].

QA is an essential metabolite of the KP and has been thoroughly researched in the brain. Microglia produce QA and serve as the primary source, as astrocytes and neurons lack the necessary machinery to produce QA; however, they can absorb it to produce NAD [37]. It has been demonstrated that neurons in the hippocampus, striatum, and neocortex are sensitive to quinolinic acid (QA), while neurons in the cerebellum and spinal cord are less responsive. These regional sensitivity differences likely relate to variations in the NMDA receptor configuration. QA is a weak endogenous agonist of NMDARs, and there is ample evidence that, at high concentrations (>100–300 nM), it is associated with neurotoxicity [68]. For instance, overstimulation of NMDARs leads to a phenomenon known as excitotoxicity, increasing glutamate release and hindering its uptake by astrocytes, inhibiting glutamine synthetase and resulting in excessive glutamate accumulation in the microenvironment and neurotoxicity [69] (Figure 2). QA has also been shown to induce lipid peroxidation by interacting with Fe_2_^+^ and forming QA–Fe_2_^+^ complexes that promote the generation of reactive oxygen species (ROS) [70,71,72]. Additionally, QA has been reported to upregulate the expression of IL-8, CCL-5, and MIP-1, as well as receptors (CXCR4, CXCR6, CCR3, and CCR5) that promote chemotaxis to the CNS [73]. QA has further been reported to induce apoptosis in astrocytes, disrupt astroglial function, cause glucotoxicity [74], increase nitric oxide production [75,76], and elevate the phosphorylation of structural proteins and tau [77,78].

### 1.3. AD and The KP

Regarding the KP and AD, it has been reported that the enzyme IDO1 is expressed in the hippocampus and structures involved in AD. For example, samples from patients with AD showed an increase in immunoreactivity of the IDO1 enzyme with the presence of senile plaques [79]. Specifically, IDO1 is localized near neurofibrillary tangles in hippocampal sections of post-mortem AD patient brains [80]. It has also been shown that Aβ42 induces the expression of IDO1 in microglia and astrocytes [81,82]. Moreover, a recent study revealed that Aβ and tau oligomers increase Kyn production by activating IDO1 in astrocytes in vitro and in vivo. Kyn then binds and translocates AhR to the nucleus, where the AhR/ARNT complex triggers gene transcription and suppresses astrocytic glucose metabolism. Conversely, inhibiting IDO1 activates AhR/HIF1α and leads to the transcription of glycolytic genes, restarting glucose metabolism and lactate production in astrocytes [82] (Figure 2), which is essential for neuronal mitochondrial respiration and synaptic activity [83].

TDO has been detected in the hippocampus in both a transgenic AD model and humans, co-localizing with QA, NFTs, and amyloid deposits [84]. On the other hand, using AD mouse models [APOE knockout and APPswe/PSEN1dE9 double-transgenic (APP/PS1)], Duan Z. et al. demonstrated that proinflammatory cytokines and KP enzymes (IDO1, KYNU, KMO, and HAOO) were upregulated in the mouse hippocampus [85].

Several studies have measured KP metabolite concentrations in serum, plasma, cerebrospinal fluid (CSF), and urine and established a relationship with AD (Table 1). For instance, the Kyn/Trp ratio detected in different bodily fluids may indicate an increased risk of developing AD and potentially serve as a valuable biomarker to identify KP-related metabolic disorders; in other words, the risk increases as more Trp is converted into Kyn, leading to an elevated Kyn/Trp ratio. It, in turn, implies an increase in systemic Kyn that can cross the BBB, resulting in high concentrations of Kyn in the brain, which are reflected in the elevated levels of other metabolites in CSF, serum, plasma, and urine [40].

On the other hand, two meta-analysis studies reported that Trp levels in the blood are significantly lower in AD patients than in controls [86,87]. Additionally, blood levels of Kyn, KA, AA, and 3-HK were not significantly different between the groups studied [86]. A different meta-analysis study found that Trp levels were decreased only in peripheral blood. The Kyn/Trp ratio was only increased in the peripheral blood of the AD group. 3-HK levels were only reduced in the CSF. KA was increased in the CSF and decreased in peripheral blood [88]. In Europe, a comprehensive multicenter investigation was conducted to analyze metabolic profiles of patients clinically diagnosed with AD and mild cognitive impairment (MCI) using samples of urine and serum stored in the AddNeuroMed/Dementia Case Register biobank across Europe. This study reported significantly lower levels of Kyn (in serum), KA (in urine), Trp (in urine and serum), and the K/T ratio (in urine) in individuals with AD compared to the control group [89]. Moreover, a recent study of AD patients reported significant positive correlations between serum and CSF concentrations of Kyn (r = 0.74), as well as between CSF levels of Tpr and 5-OH-Tpr [5-hydroxy-l-tryptophan (r = 0.74)], Kyn and KA (r = 0.79), and 5-OH-IAA and IAA [indole-3-acetic acid (r = 0.77)] [90]. Another case–control study using CSF samples from AD patients reported increased KA and PA concentrations (controls: 2.8 and 20.7 nM; cases: 3.6 and 23.2 nM, respectively) [91]. Similarly, other studies have reported increased levels of KA in the CSF of AD patients [92,93], which could be specific to this disease since it has not been observed in other neurodegenerative diseases [94]. Correlation studies have also been conducted between neurodegeneration biomarkers (neurofilament light chain; NFL), AD amyloid pathology, and KP metabolites. Chatterjee P. et al. demonstrated a positive correlation between NFL and various KP metabolites (Kyn, KA, 3-HK, AA, and QA) in blood samples and a positive correlation between Aβ and the metabolites, as mentioned above. It suggests an association between neurodegeneration and neuroinflammation in AD [95]. Elevated Trp, KA, and QA concentrations have also been associated with Aβ142, tau, and p-Tau-181 in CSF [92].

**Table 1:** Overview of studies examining the enzymes and metabolites of the KP in different tissues and their connection to AD.

Sample	Change	Observations	References
Hippocampus Human primary macrophages and microglia Primary mouse astrocytes	IDO1 overexpressed	IDO1 inhibition reduced Aβ neurotoxicity. IDO1 and Kyn suppress astrocytic glucose metabolism.	[80,81,82,83]
Human and transgenic mouse hippocampus	TDO overexpressed QA increased	TDO and QA co-localized with NFTs and Aβ plaques	[84]
Transgenic mouse hippocampus	IDO1, KMO, and HAOO overexpressed	Upregulated in the mouse hippocampus	[85]
Serum or plasma	SMD = −0.68 Trp	Trp blood levels were significantly lower in the blood of AD patients.	[86]
Serum or plasma	SMD = −0.520 Trp	Significant decrease in Trp in AD patients. There is a significant difference in the Kyn/TRP ratio.	[87]
Serum or plasma CSF	SMD = −0.82 Trp SMD = −0.35 KA SMD = 0.54 Kyn/Trp SMD = −1.17 3-HK SMD = 0.70 KA	Trp and KA blood levels were significantly lower in AD. Elevated Kyn/Trp ratio associated with increased AD risk. 3-HK decreased and KA increased in CSF	[88]
Urine Serum	Trp Kyn KA	Lower levels of Kyn (in serum), KA (in urine), Trp (in urine and serum), and the K/T ratio (in urine) in individuals with AD compared to the control group.	[89]
CSF	(C) 4.26 nM KA (AD) 5.5 nM KA	KA showed significantly higher concentrations in CSF of AD patients.	[91]
CSF	(C) 2.8 nM KA (AD) 3.5 nM KA (C) 20.7 nM PA (AD) 23.2 nM PA	The patients with AD showed higher concentrations of KA and PA compared to the control group.	[92]

SMD, standardized mean difference; C, control; CSF, cerebrospinal fluid.

### 1.4. AD and AhR

AhR is a xenobiotic (compound external to the organism) and endobiotic receptor that regulates various cellular processes. This receptor is activated by the binding of the following: ligands derived from environmental substances like dioxins and dioxin-like compounds [96]; ligands derived from the diet (cruciferous vegetables), such as indole-3-carbinol (I3C) [97]; derivatives from the gut microbiota, such as indole-3-acetic acid (IAA), tryptamine (TA), and 3-methylindole [98]; and ligands originating from Trp metabolism, such as Kyn and KA [99]. Recently, endogenous agonists derived from Trp degradation by IL4-induced L-amino acid oxidase (IL4I1) have been reported, such as indole-3-aldehyde (I3A) and KA [100]. AhR is inactive in the cellular cytoplasm, complexed with various proteins (HSP90, AIP, PTGES3, and SRC). Upon exposure to its ligand, it translocates to the nucleus and forms a complex with the AhR nuclear translocator (ARNT), as mentioned previously. This complex is a transcription factor for genes containing xenobiotic response elements (XERs) [101]. Concerning its expression in the brain, AhR is expressed in various cell types, including neurons, astrocytes, and microglia, particularly in the brainstem and the hippocampus [102].

Some research has indicated possible connections between AhR and AD, and the exact nature of these links is still being explored. Since neuroinflammation is linked to aging and related to the onset of AD, a comparative study reported an increase in AhR expression in the brains of older individuals and AD patients, especially in astrocytes [103]. Recently, it was demonstrated that AhR was activated in the hippocampus of AD patients [85]. On the other hand, it was determined that in HT22 cells (derived from mouse hippocampus), exposure to different concentrations of Aβ led to increased expression of IDO1 and AhR, and phosphorylation of β-catenin (an inactive pathway marker) and tau, but decreased phosphorylation of GSK3β. With the previous one, it was demonstrated that the Wnt/β-catenin pathway is inactive in AD and that it depends on the Aβ-IDO1-Kyn-AhR-Dickkopf-1 axis (the latter inhibits the Wnt/β-catenin pathway), increasing tau phosphorylation [104].

The role of Neprilysin (NEP), the primary enzyme responsible for degrading Aβ in both the brain and periphery, has also been studied [105,106]. NEP expression and activity decrease during aging and AD progression, possibly contributing to its development [107]. On the other hand, increasing NEP in murine AD models has been reported to mitigate the effects of the disease [108]. Regarding the relationship between NEP, AhR, and AD, it was demonstrated that AhR activation by Diosmin, Kyn, or I3C directly induces NEP expression, reducing Aβ levels and decreasing cognitive deficits in AD patients [109].

**Figure 3:**
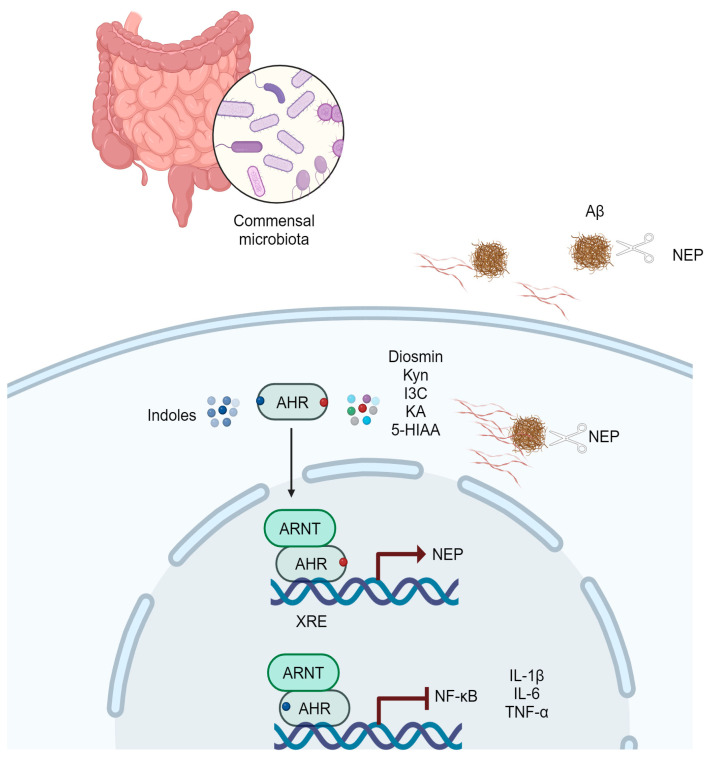
AHR activation and neuroprotection in AD. Ligands such as diosmin, Kyn, I3C, KA, and 5-HIAA activate AHR and induce the expression of NEP, a metalloendopeptidase that contributes to the degradation of Aβ plaques [109]. Indoles derived from the commensal microbiota activate the AHR and suppress the expression of NF-κB, thereby reducing the production of proinflammatory cytokines (IL-1β, IL-6, TNF-α) [113]. In AD, NEP expression and activity decrease. Furthermore, dysbiosis minimizes the production of indoles derived from commensal microbiota. Kyn, kynurenine; I3C, Indole-3-carbinol; KA, kynurenic acid; 5-HIAA, 5-hydroxyindole-acetic acid; NEP, Neprilysin; ARNT, AHR, Aryl hydrocarbon receptor; AHR nuclear translocator; XRE, xenobiotic response element.

Recent research suggests that gut microbiota dysbiosis may influence the development and progression of AD [110,111]. According to the above-mentioned, a decrease in the abundance of indole-producing intestinal bacteria in AD patients [87] and diminished microbial indole metabolites (5-Hydroxyindol, Indole-2-carboxylic acid, 3-(2-Hydroxyethyl) indole) was reported [112]. Moreover, using the APP/PS1 mouse model, it was reported that microbiota-derived indoles activate AhR and inhibit neuroinflammation [113] (Figure 3); Additionally, a high-Trp diet markedly ameliorates cognitive dysfunction, reduces Aβ depositions, and activates AhR [114].

Defects in neurogenesis of neural stem cells related to AD have been described [115,116]. For example, it has been observed that Aβ42 increases the proliferation of neural stem cells [116]. Recently, using the zebrafish as an AD model, it was shown that the metabolite KA reduces the proliferation of neural stem cells through AhR2 signaling; conversely, Aβ-induced neuropathology suppresses tryptophan metabolism through IL4 [117].

## 2. Discussion

The present review provides evidence about the connection between the KP, AhR activation, and AD. The KP is an important metabolic pathway that produces several metabolites thought to be involved in neurodegenerative diseases. This pathway is activated in response to systemic inflammation and neuroinflammation, allowing for the conversion of tryptophan into these metabolites and, therefore, playing a key role in regulating inflammatory processes. In addition to inflammatory responses, these metabolites are also involved in neurotransmission, neuronal growth, synaptogenesis, and other functions [118].

This manuscript highlights research on altered levels of KP metabolites in biological samples, including serum, plasma, and CSF from AD patients; however, there are controversial findings. Recently, Knapskog et al. reported higher concentrations of the neuroprotector metabolite KA in CSF, suggesting that the activation of the KP’s neuroprotective branch may be an adaptive response in AD [91]. Similarly, a meta-analysis review reported that levels of KA were increased in CSF but diminished in blood [88]. In contrast, other authors have reported reduced or no change in KA levels in CSF and plasma [119,120]. These variations might be accounted for by the absence of detailed information in the studies regarding the specific AD stage, patients’ treatment details, cognitive deficits, and psychiatric comorbidities. Furthermore, it should be considered that not all KP metabolites cross the BBB.

It is crucial to emphasize that reports indicate that levels of the neurotoxic metabolite QA remain consistent between control subjects and individuals diagnosed with AD [87,88]. However, a hypothesis suggests that QA may accumulate in the brain and be associated with tau phosphorylation [78]. Therefore, additional investigations involving CSF and peripheral blood (including plasma and serum) are required to arrive at more robust conclusions.

Given the activation of AhR by specific metabolites of the KP, our focus involved seeking evidence regarding its association with AD. However, until now, there has been limited exploration of the expression and functionality of AhR in AD. Nevertheless, AhR expression and activation in the hippocampus of mice with AD were recently observed [85], suggesting that activation of AhR elicits pathological reactions linked to AD. In contrast, AhR activation is associated with a high-Trp diet [114], a NEP-dependent decrease in Aβ levels, and diminishing cognitive deficits in AD patients [109]. This inconsistency could be elucidated by considering the nature of the ligand engaging the AhR. Various substances originating from the metabolic pathways of Trp, such as 5-hydroxyindole-acetic acid (5-HIAA, serotonin pathway) and kynurenic acid (KA), have been recognized as modulators of NEP expression and functionality [108,121]. These substances play a role in mitigating cerebral toxicity resulting from the accumulation of amyloid-beta (Aβ) by promoting the degradation of Aβ. On the other hand, indoxyl sulfate is a potent AhR activator [122], and it is established that indoxyl sulfate can compromise the integrity of the blood–brain barrier through the activation of AhR [99] and could enhance the pathogenesis of AD.

With little to no cure imminent, there is a need to explore innovative approaches to understanding and challenging AD. In recent years, research has started investigating the role of the KP and the potential interplay concerning AD [123]. IDO1 is an essential enzyme in the KP, and in the context of AD, the activation of IDO1 is regarded as a pathogenic determinant of inflammation associated with Aβ [124]. Therefore, using IDO1 as a therapeutic target will decrease the accumulation of harmful intermediates in KP, oxidative stress, cytotoxicity, and tau phosphorylation. However, several IDO1 inhibitors used as immunotherapies in tumors, unfortunately, exhibit low lipophilicity and inefficient brain penetration [125].

While research into the interplay between AD and the KP is still in its beginnings, it has the potential to provide new insights into both the pathophysiology of AD and novel therapeutic strategies for this disorder. As knowledge about this complex network grows, further research will be necessary to elucidate its role in AD and develop effective treatments. Studying biomarkers in peripheral fluids like serum or urine using metabolomic and bibliometric analyses can provide valuable information about the metabolic changes associated with AD.

Finally, further studies on animal models or clinical trials are needed to validate current findings and develop more effective treatments for AD.

## 3. Conclusions

The KP is the primary route for the degradation of tryptophan, and disturbances in this pathway have been linked to neurodegenerative processes like AD. Metabolites originating from this pathway are found at elevated levels in samples from AD patients. However, it is essential to note that not all metabolites from the KP are associated with AD. High levels of the metabolite QA have been primarily associated with neurotoxicity and AD. Furthermore, metabolites derived from Tpr metabolism, particularly indoxyl sulfate, a potent AhR inducer, have been associated with numerous toxic effects related to AD. This underscores the complexity of the relationship between tryptophan metabolism, AhR activation, and AD, with both neuroprotective and detrimental effects potentially stemming from the same pathway. Further research is required to elucidate the precise mechanisms and the net impact of these intricate interactions in the development and progression of AD.
